# Supplementary material for: Fast plasmoid-mediated reconnection in a solar flare
Source: Nat Commun. 2022 Feb 2;13:640. doi: 10.1038/s41467-022-28269-w (PMC8810921; doi:10.1038/s41467-022-28269-w)
Supplement: Supplementary file 1 — supplementary information [file 41467_2022_28269_MOESM1_ESM.pdf]

Supplementary Information
Fast plasmoid-mediated reconnection in a solar flare

Xiaoli Yan[1,2,3], Zhike Xue[1,3], Chaowei Jiang[4], E. R. Priest[5], Bernhard Kliem[6], Liheng Yang[1,3], Jincheng Wang[1,3], Defang Kong[1,3], Yongliang Song[7], Xueshang Feng[2], Zhong Liu[1,8]

December 14, 2021

[1]Yunnan Observatories, Chinese Academy of Sciences, Kunming, Yunnan 650216, China.(email: yanxl@ynao.ac.cn)
[2] State Key Laboratory of Space Weather, Chinese Academy of Sciences, Beijing 100190, China.
[3] Center for Astronomical Mega-Science, Chinese Academy of Sciences, 20A Datun Road, Chaoyang District, Beijing, 100012, China.
[4] Institute of Space Science and Applied Technology, Harbin Institute of Technology, Shenzhen, 518055, China.(email: chaowei@hit.edu.cn)
[5] School of Mathematics and Statistics, University of St Andrews, St Andrews, KY16 9SS, UK.
[6] Institute of Physics and Astronomy, University of Potsdam, Potsdam 14476, Germany.
[7] Key Laboratory of Solar Activity, National Astronomical Observatories, Chinese Academy of Sciences, Beijing 100012, China.
[8] University of Chinese Academy of Sciences, Yuquan Road, Shijingshan Block, Beijing 100049, Peoples Republic of China.

1 Supplementary Figures

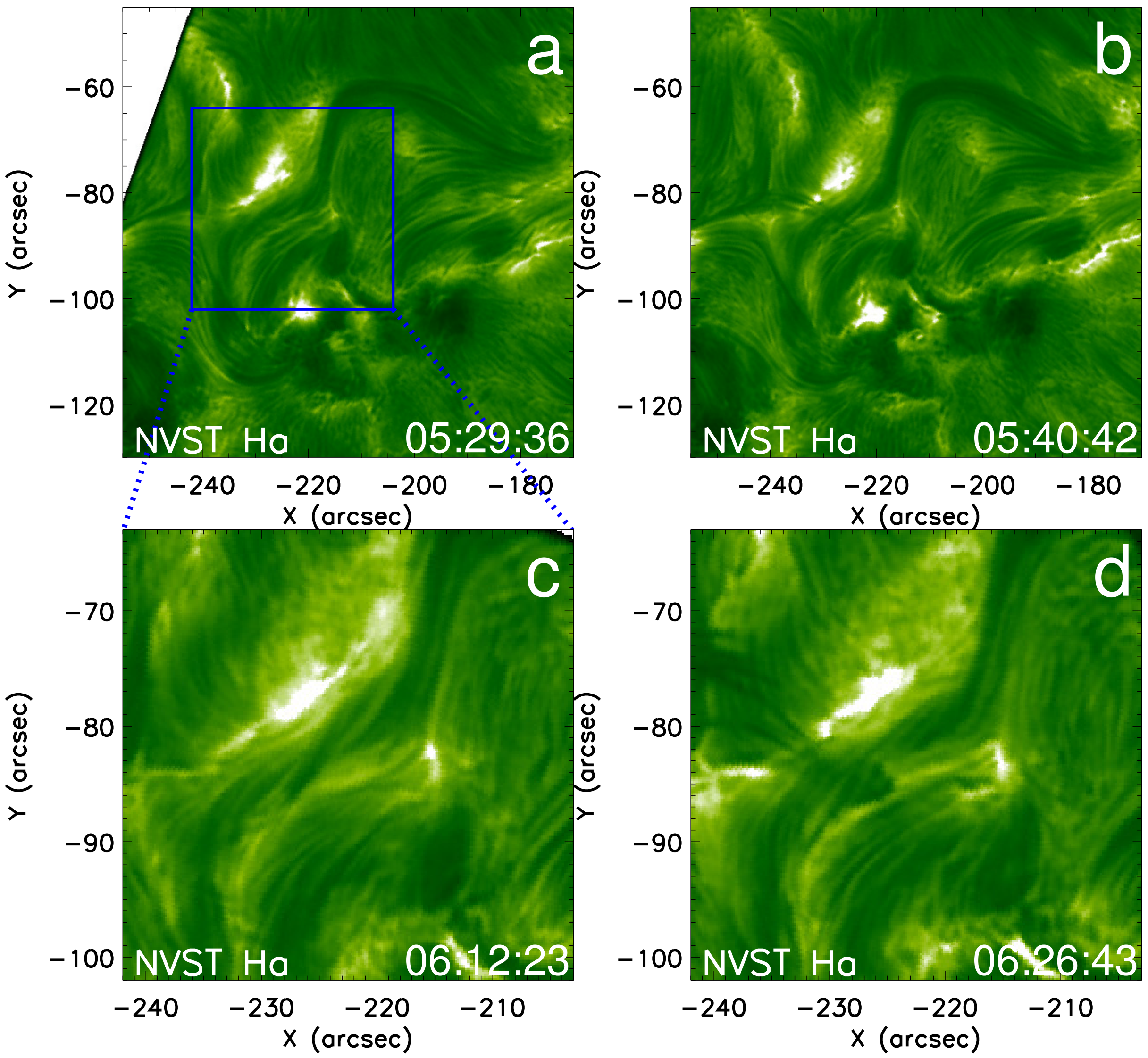

Supplementary Figure 1. The structure of the filament in the Hα images observed by the NVST. (a, b): The whole structure of the filament in the Hα images at 05:29:36 UT and 05:40:42 UT, respectively. (c, d): High twist part of the filament in the Hα images at 06:12:23 UT and 06:26:43 UT, respectively. The threads of the filament are winding each other, which supports that the magnetic structure of the filament is a flux rope.

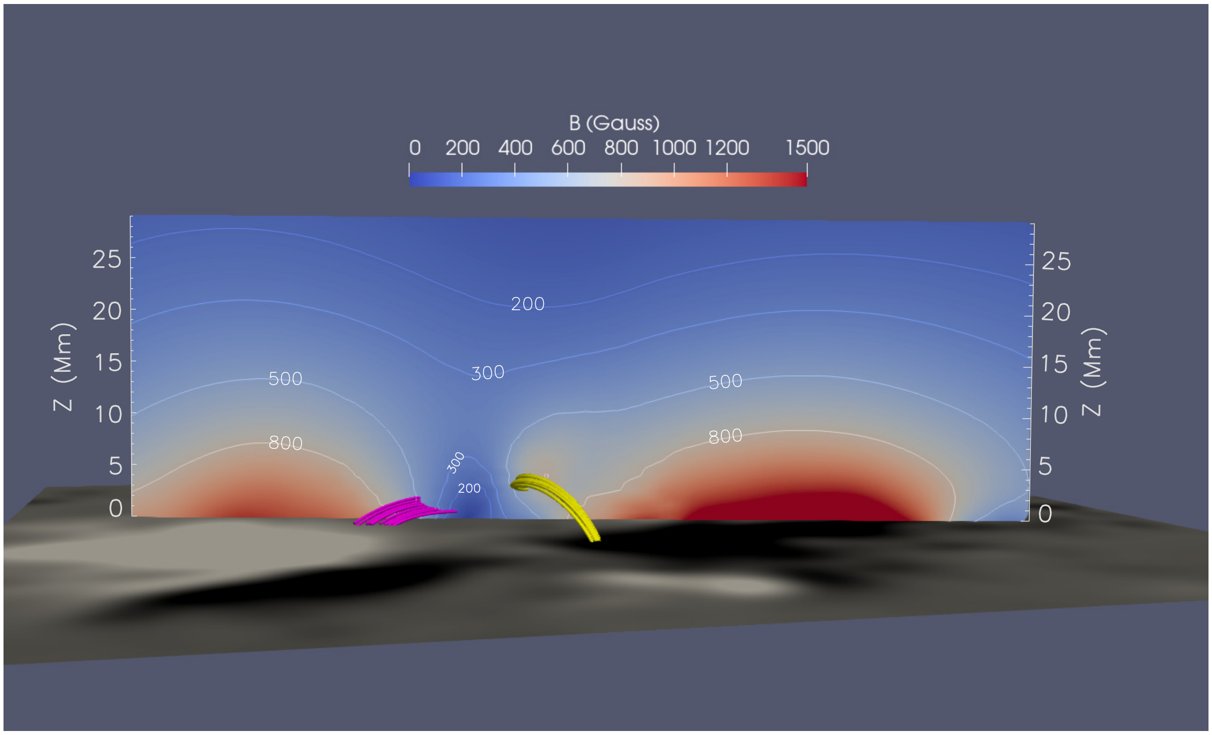

Supplementary Figure 2. Magnetic field strength in the cross-section of the NLFFF through the reconnection region. The selected yellow lines and the pink lines show the same structures as in Figure 1b. The different colors show the different magnetic field strength in the cross-section. The white contours outline the regions of the different magnetic field strength. The axial field in the cross section of the rope exceeds 500 G in the height range $z \sim 5$–15 Mm. Note that the z-axis denotes the height perpendicular to the x-y plane.

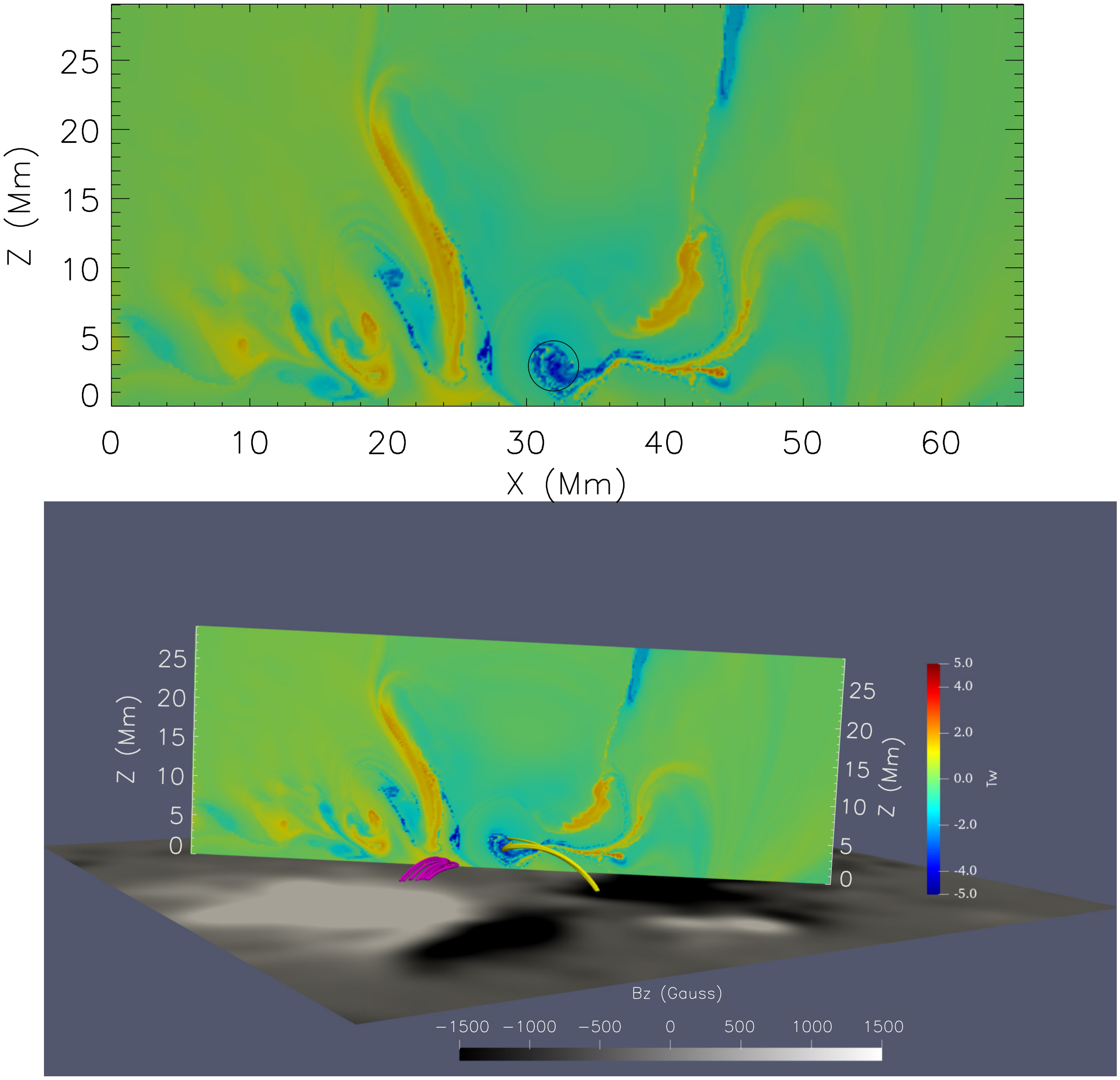

Supplementary Figure 3. Twist (T_{w})[1] in the same cross section. The selected yellow lines and the pink lines show the same structures as Figure 1b. The different colors show the different values of twist. The circle in the upper panel indicates the cross section that is used to represent the radius of the strongly twisted core of the flux rope **in Eq. (2)**. Note that the z-axis denotes the height perpendicular to the x-y plane.

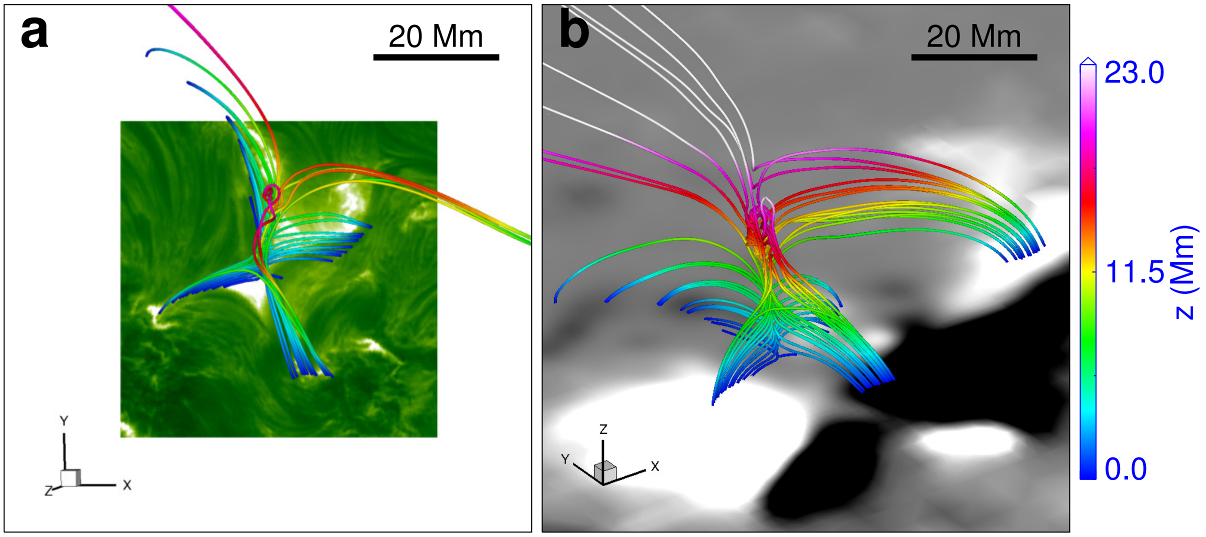

Supplementary Figure 4. Magnetic connections between the coronal current sheet and chromospheric flare ribbons. (**a**) Field lines in the MHD model at $t = 42$ s show that the coronal current sheet in the range of significant reconnection ($z \sim 3$–15 Mm, cyan to red colours) is magnetically connected to the observed chromospheric flare ribbons as seen in Hα at 08:09:21 UT. The field lines connecting the higher layers in the current sheet with more remote locations spread out more, which implies a lower density of energy deposition from the current sheet to the chromosphere, i.e., a lower likelihood of ribbon formation. (**b**) Perspective view including the longitudinal magnetogram at 08:00 UT.

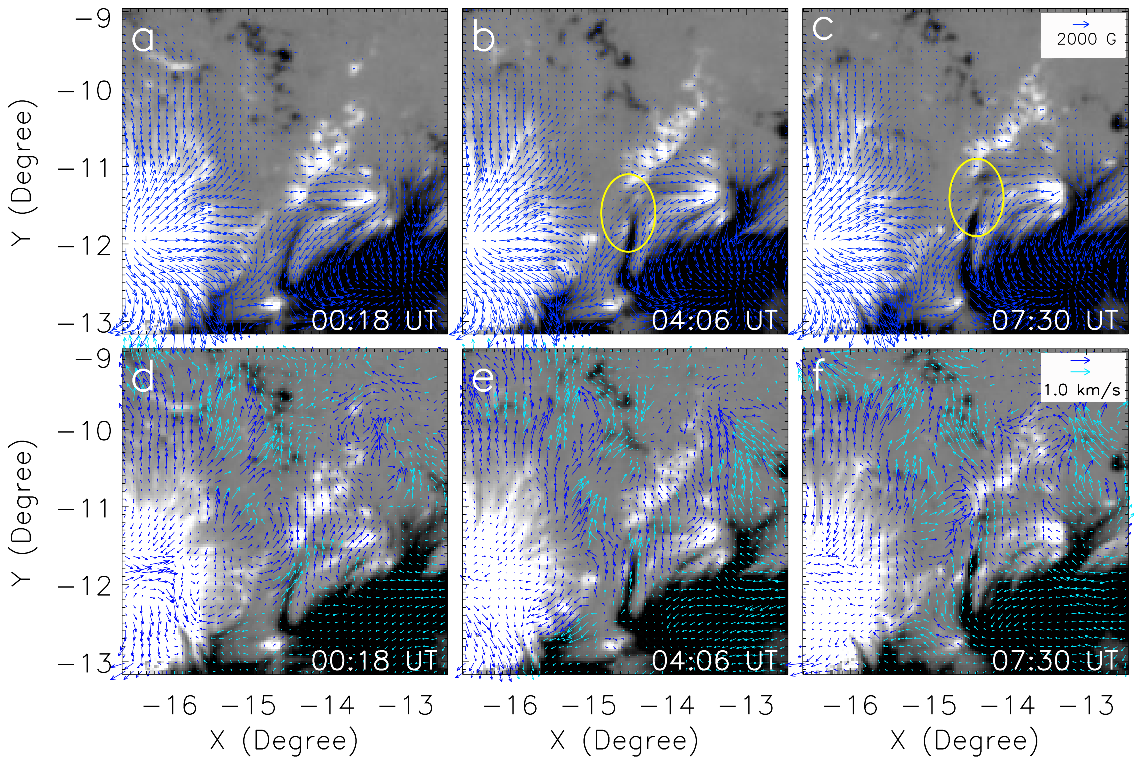

Supplementary Figure 5. Evolution of the photospheric vector magnetic field observed by SDO/HMI and flow field derived using the DAVE algorithm. (a–c) Vector magnetograms. The greyscale images show the vertical field component, saturated at ± 1000 G, and the blue arrows show the horizontal component. The length of the blue arrow in the white box denotes the magnetic field strength of 2000 G in panel c. The yellow ellipses denote the position of magnetic emergence. (d–f) Evolution of the flow field. Blue and cyan arrows indicate the horizontal photospheric velocity in the positive and negative polarities, respectively. The length of the blue and cyan arrows in the white box denotes the velocity of 1.0 km s^{-1} in panel f. Note that the east and west directions described in the text correspond to the left and the right sides of the Figures.

Supplementary References

[1] Berger, M. A. & Prior, C. The writhe of open and closed curves. *Journal of Physics A Mathematical General* **39**, 8321–8348 (2006).
