# Supplementary material for: Fast plasmoid-mediated reconnection in a solar flare
Source: Nat Commun. 2022 Feb 2;13:640. doi: 10.1038/s41467-022-28269-w (PMC8810921; doi:10.1038/s41467-022-28269-w)
Supplement: Supplementary file 3 — Description of Additional Supplementary Files [file 41467_2022_28269_MOESM3_ESM.docx]

**Description of Additional Supplementary Files**

**File Name:** Supplementary Movie 1

**Description:** The structure of the filament and the process of the magnetic reconnection in the Hα images observed by the NVST.

**File Name:** Supplementary Movie 2

**Description:** SDO/AIA EUV and UV images and simultaneous evolution of the SDO/HMI line-of-sight magnetogram during the occurrence of the M-class flare.

**File Name:** Supplementary Movie 3

**Description:** SDO/AIA 211 Å images processed with the unsharp masking technique to show the motion of blobs (plasmoids and mini flux ropes) along the current sheet and separatrices.

**File Name:** Supplementary Movie 4

**Description:** Animated views of the MHD simulation state immediately before the onset of reconnection. Top left: magnetogram, reconnecting field, and current density isosurface, color coded with height information. Top right: magnetogram, reconnecting field, and separatrices or QSLs (iso-surface of squashing degree, log10 Q = 3), color coded with height information. Bottom left: horizontal cuts of the current density in the relevant height range of the reconnection process. Bottom right: Same for the squashing degree.

**File Name:** Supplementary Movie 5

**Description:** Evolution of the photospheric magnetic and velocity field of Active Region NOAA 11967 on 2014 February 2. The SDO/HMI vertical field component, saturated at 1000 G, is shown in grey scale and the inferred horizontal velocities are represented by the arrows.
